# Association of *PSORS1C3*, *CARD14* and *TLR4* genotypes and haplotypes with psoriasis susceptibility

**DOI:** 10.1590/1678-4685-GMB-2022-0099

**Published:** 2022-11-07

**Authors:** Nguyen Thi Thuy Linh, Nguyen Hoang Giang, Nguyen Thi Kim Lien, Bui Kieu Trang, Do Thi Trang, Nguyen Thy Ngoc, Vu Xuan Nghia, Le Tra My, Can Van Mao, Nguyen Huy Hoang, Nguyen Thi Xuan

**Affiliations:** 1 University of Science and Technology, Vietnam Academy of Science and Technology, Ha Noi, Vietnam.; 2 Institute of Genome Research, Vietnam Academy of Science and Technology, Ha Noi, Vietnam.; 3 University of Science and Technology of Hanoi, Vietnam Academy of Science and Technology, Ha Noi, Vietnam.; 4 108 Military Central Hospital, Hanoi, Vietnam.; 5 Vietnam Military Medical University, Department of Pathophysiology, Ha Dong, Hanoi, Vietnam.

**Keywords:** CARD14, polymorphism, PSORS1C3, psoriasis, TLR4

## Abstract

Psoriasis is a common chronic, immune-mediated inflammatory disease of the skin. *PSORS1C3* is a non-protein coding gene, of which the RNA transcript is found in psoriatic patients. CARD14 is mainly expressed in epidermal keratinocytes. TLR4 is a transmembrane protein to recognize microbial antigens. Our study aimed to assess the relationship among *PSORS1C3, CARD14* and *TLR4* polymorphisms, inflammatory expression and psoriasis susceptibility. To the end, 71 patients with psoriasis and 46 healthy individuals with the well-characterized clinical profiles were enrolled. Gene polymorphisms were determined by Sanger DNA sequencing and secretion of cytokines by ELISA. As a result, genetic analysis of *PSORS1C3* gene identified nine SNPs and three haplotype blocks. Sequencing of the *CARD14* gene determined eight SNPs and one haplotype block. Sequencing of *TLR4* gene identified nine SNPs, in which a SNP rs1018673641 was found to exert deleterious effect. The linkage disequilibrium analysis showed that seven variants in *PSORS1C3* gene and three SNPs in *CARD14* gene were in tightly linked. More importantly, a significant association between IL-6 level and rs1018673641 AT genotype in *TLR4* gene was detected in psoriatic patients. In conclusion, the *PSORS1C3, CARD14* and *TLR4* polymorphisms and haplotypes may be correlated with risk of suffering psoriasis and the IL-6-mediated chronic inflammation in psoriasis could be partially regulated by the *TLR4* functional variant.

## Introduction

Psoriasis is a common chronic, immune-mediated inflammatory skin disease, affecting 2-3% of the population (Afonina et al., 2021). The typical clinical manifestation of psoriasis includes widely distributed scaly erythema or plaques, which are commonly located on the scalp, elbows, knees and lumbar area (Parisi et al., 2013). There are five different types of psoriasis, in which plaque psoriasis is the most common form of the disease and accounts for approximately 90% of the cases (Griffiths *et al.,* 2007). Psoriasis is associated with systemic inflammation caused by the recruitment of inflammatory cells, including macrophages, T cells and neutrophils into the epidermis of psoriatic skin (Takeshita et al., 2017; Ren et al., 2020). Individuals with psoriasis are at an increased risk of developing other serious diseases, such as hypertension, diabetes, liver and kidney disease, and atherosclerosis (Takeshita *et al.,* 2017). During the inflammatory process, activation of Th1 and Th17 cells via a nuclear factor kappa-light-chain-enhancer of activated B (NF-κB) pathway is triggered by mature plasmacytoid and myeloid dendritic cells, which are migrated into the skin-draining lymph nodes and produce psoriasis relevant mediators of inflammation (Wagner et al., 2010; Mease, 2015; Tang et al., 2021). 

Genetic investigations have shown that psoriatic patients have diverse gene polymorphisms related to skin barrier function (van den Bogaard et al., 2014). More than 30 single nucleotide polymorphisms (SNPs) have been associated with the risk of developing psoriasis. Among them, the psoriasis susceptibility 1 (*PSORS1*) gene is the major susceptibility locus for psoriasis (Mohd Affandi et al., 2018). PSORS1 is located within the major histocompatibility complex (MHC) on chromosome 6p21.3 (Mohd Affandi *et al.,* 2018), in which the non-protein coding gene *PSORS1C3* is an established susceptibility gene for psoriasis and its RNA transcript is found both in psoriatic patients and control individuals (Holm et al., 2005). The -26C and +246A alleles in the *PSORS1C3* gene are strongly associated with psoriasis in the Swedish, but not in Chinese population (Holm *et al.,* 2005). Although the association of *PSORS1C3* polymorphisms with psoriasis has been widely studied (Holm *et al.,* 2005; Chang et al., 2006), the regulatory role of *PSORS1C3* on immune cell function is little known.

Immunogenomics studies have indicated that constitutive activation of toll-like receptors (TLRs) is induced by their abnormal mRNA expression or causal mutations (Smith et al., 2016; Shao et al., 2019). Each of the TLR subtypes has its own specific recognition pattern upon ligand engagement, in which TLR4 is the transmembrane protein expressed on antigen-presenting cells to recognize microbial antigens and contributes as the initiator of inflammatory response in innate immunity (Traks et al., 2015). A recent study reveals that in patients with psoriasis, activation of the NF-κB signaling is triggered by TLR4 (Tang et al., 2021) to exert chronic inflammatory condition (Takeshita et al., 2017). The release of inflammatory cytokines such as TNF-α/IL-23/IL-17 in psoriatic patients is involved in excessive proliferation and aberrant differentiation of keratinocytes (Mease, 2015; Takeshita *et al.,* 2017). The serum level of the cytokines is correlated with the clinical severity and pathogenesis of psoriasis (Cai et al., 2019). Polymorphisms within the *TLR4* gene have been associated with a number of immune-mediated inflammatory diseases, such as psoriasis (Smith *et al.,* 2016). *TLR4* expression is increased in the epidermis of psoriatic skin, but weak in healthy controls (Shao *et al.,* 2019). Role of TLR4 signaling is also of special importance in regulating psoriasis-like inflammation in patients with kidney failure (Ren et al., 2020). In mice, activation of TLR4 results in the release of pathogenic cytokines including IL-17 in the mouse model of arthritis (Chovanova et al., 2013). The constitutive IL-1β activation leads to a Th17-mediated response and a psoriatic phenotype (Shepherd et al., 2004).

Similar to TLR4, mutations in the *caspase recruitment domain-containing protein 14* (*CARD14*) gene are associated with immune response in psoriatic patients via NF-κB signaling (Scudiero et al., 2011) to exert the release of key psoriatic chemokines, such as CCL20 and CXCL8/IL-8 (Lowes et al., 2014). CARD14 is an intracellular scaffold protein and mainly expressed in epidermal keratinocytes. Genetic variants in the *CARD14* gene have been shown to increase NF-κB activity in keratinocytes of psoriatic patients (Danis et al., 2018). Mouse keratinocytes lacking *CARD14* produce reduced IL-17A due to the inactivation of NF-κB and mitogen-activated protein kinase (MAPK) pathways (Wang et al., 2018). 

Investigations on the effects of *PSORS1C3, CARD14,* and *TLR4* polymorphisms are gradually increasing in the field of biomarker study in inflammatory diseases, such as psoriasis (Holm et al., 2005; Scudiero et al., 2011; Smith et al., 2016). In this study, SNP profiling of *PSORS1C3, CARD14,* and *TLR4* genes in 71 patients with psoriasis and 46 healthy individuals by direct DNA sequencing was investigated to determine disease-associated SNPs. Besides, inflammatory expression of psoriatic patients was also assessed by enzyme-linked immunosorbent assay (ELISA) to determine the relationship between serum level of cytokines and the presence of unusual genotypes.

## Material and Methods

### Patients and control subjects

A total of untreated 71 psoriatic patients and 46 healthy volunteers used as controls were recruited into the study at the Thien Phu Duong Traditional Medical Clinic, Hanoi, Vietnam. The diagnosis of psoriasis was based on the 2016 WHO criteria (World Health Organization., 2016), including sharply demarcated round-oval erythematous plaques with loosely adherent silvery-white scales, especially affecting the elbows, knees, lumbosacral area, intergluteal cleft, and scalp. No individuals in the control population took any medication or suffered from any known acute or chronic disease. All patients and volunteers gave a written consent to participate in the study. Person care and experimental procedures were performed according to the Vietnamese law for the welfare of humans and were approved by the Ethical Committee of the Institute of Genome Research, Vietnam Academy of Science and Technology and all experimental protocols on human subjects were in accordance with Helsinki Declaration of 1975, as revised in 2008.

### Sample size calculation and power of study

This study was designed to demonstrate 40% mean difference with 80% power and 5% significance level, a sample size of 40 in each group was calculated as described elsewhere (In et al., 2020). To allow for study error and attrition, 71 psoriatic patients and 46 healthy individuals were included in this investigation. 

### DNA sequencing of PSORS1C3, CARD14 and TLR4

Genomic DNA was isolated from peripheral blood samples using a DNeasy blood and tissue kit (Qiagen). To determine polymorphisms of the *PSORS1C3, CARD14* and *TLR4* genes, polymerase chain reaction (PCR) and DNA sequencing (3500 Genetic Analyzers, Thermo Scientific) were performed as previously described (Trang et al., 2022). The GenBank accession numbers AY484516.1, NM_001366385.1 and NM_138554.5 were used for DNA sequence analysis of *PSORS1C3, CARD14* and *TLR4*, respectively, by using primers: PSORS1C3-F: 5’- TTTGGATGTGTCAGATTTAAGGCC-3’ and PSORS1C3-R: 5’- AATAACGAATGCAGCTGCACAT-3’; CARD14 -F: 5’- CTGCAGTGAGCAAAGCAGAC-3’ and CARD14-R: 5’- CAGGTGAGTGTGGGAATGTG-3’; and TLR4-F: 5’- TTGGTCCACAACGGTTCTCTG-3’ and TLR4-R: 5’- CTGGATGGGGTTTCCTGTCA-3’. The amplification product lengths of *PSORS1C3, CARD14* and *TLR4* were 665, 399 and 737bp, respectively. All obtained PCR fragments were purified with a GeneJET PCR purification kit (Thermo Scientific). The PCR products were sequenced on both strands with the same primers used for the PCR.

### Cytokine quantification

Sera were isolated from the blood samples of psoriatic patients and healthy subjects and stored at -20 ˚C until used for ELISA. TNF-α, IL-6, and IL-17A concentrations were determined by using ELISA kits (Thermo Scientific) according to the manufacturer’s protocol.

### Data analysis

Data related to the human *PSORS1C3, CARD14* and *TLR4* genes was collected from NCBI (https://www.ncbi.nlm.nih.gov/). The information for the SNP ID of these genes was retrieved from the NCBI’s SNP database (https://www.ncbi.nlm.nih.gov/snp/). Bioedit software was used for the initial analysis of the sequences. 

To analyze the functional consequence of the SNPs in *TLR4* gene, a PolyPhen2 program (http://genetics.bwh.harvard.edu/pph2/index.shtml) was used. The PolyPhen-2 score varies from 0.0 (tolerated) to 1.0 (deleterious), in which the SNPs were designated “probably damaging”, “potentially damaging”, “benign” or “unknown”. In addition, the possible impact of the intronic SNPs on slicing was predicted by SD-Score (Ohno et al., 2018) or MaxEntScan (Jian et al., 2014) predictor programs.

### Statistical analysis

The SPSS version 20 (IBM, New York, USA) was used for statistical analysis. To examine the genotype association of control and patient groups, Fisher’s exact test was used for SNPs with expected sample sizes less than 20 and Chi-squared test for those with larger expected sample sizes. The odds ratios (OR) and 95% confidence intervals (CI) were calculated by the logistic regression analysis as described elsewhere (Szumilas et al., 2010). The difference in cytokine levels among the SNPs, control and patient groups was tested for significance using the Mann-Whitney U test. The linkage disequilibrium (LD) analysis was calculated using the R Package LDlinkR. In all statistical analyses, the level of significance was determined at the level of*p*< 0.05 and two-sided.

## Results

### Association between PSORS1C3, CARD14 and TLR4 gene polymorphisms and psoriasis

**Figure 1-:**
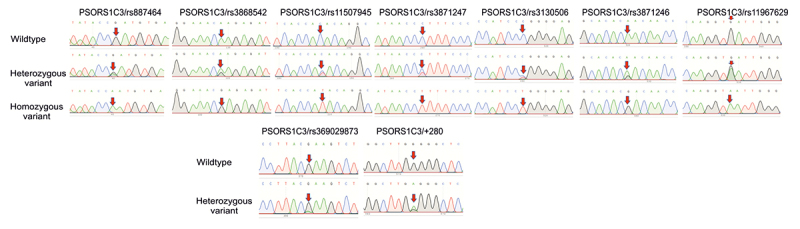
Polymorphisms of PSORS1C3 gene in psoriatic patients and controls. Partial sequence chromatograms of *PSORS1C3* gene from wildtype (1^st^ panels) and variant (2^nd^ and 3^rd^ panels) genotypes of the rs887464 G>A, rs3868542 A>G, rs11507945 C>T, rs3871247 C>T, rs369029873 G>A, rs3130506 C>T, rs3871246 A>G, rs11967629 G>A and +280 G>A polymorphisms are shown. Arrows indicate the location of the base changes.

Firstly, sequencing of the *PSORS1C3* gene identified 9 nucleotide changes including, rs887464 G>A, rs3868542 A>G, rs11507945 C>T, rs3871247 C>T, rs369029873 G>A, rs3130506 C>T, rs3871246 A>G, rs11967629 G>A and +280 G>A in the 5’ flanking region (Tables 1, 2 and Figure 1). Among the 9 SNPs found, the 4 genotypes including rs887464 AA, rs11507945 TT, rs11967629 AA and +280 GA in *PSORS1C3* gene showed significantly higher frequencies in cases (25.35%, 11.27%, 11.27% and 32.39%, respectively) compared to controls (4.35%, 0%, 0% and 13.04%, respectively). The genotype distribution of the 9 SNPs, except for the SNP rs887464 was in agreement with a Hardy-Weinberg equilibrium (HWE) (p > 0.05). In addition, we noted that the minor allele frequency (MAF) of the SNPs rs11507945, rs3871247, rs3871246, rs11967629 and +280 G>A was higher, whereas the MAF of the SNPs rs369029873 and rs3130506 was lower in cases compared to controls (Table 1). 

**Table 1 -:** General information on SNPs and haplotypes of TLR4, CARD14 and PSORS1C3 genes in psoriatic patients and controls.

SNP/Gene	Position	Type of Variant	Allele	MAF in psoriasis group	MAF in control group	1000G MAF	HWE in Psoriasis Group (p)	HWE in Control Group (p)	HWE in all Population (p)
*PSORS1C3/rs887464*	6:31178143	5′ Flanking	G>A	0.4014	0.3043	0.3722	0.0053	0.2895	0.1897
*PSORS1C3/rs3868542*	6:31178062	5′ Flanking	A>G	0.3803	0.3696	0.41154	0.3880	0.9841	0.6268
*PSORS1C3/rs11507945*	6:31177978	5′ Flanking	C>T	0.3239	0.2500	0.21705	0.9567	0.0777	0.6270
*PSORS1C3/rs3871247*	6:31177925	5′ Flanking	C>T	0.3873	0.3370	0.41154	0.5017	0.3410	0.9970
*PSORS1C3/rs369029873*	6:31177834	5′ Flanking, exon 1	G>A	0.1690	0.2065	0.00002	0.2303	0.2105	0.0516
*PSORS1C3/rs3130506*	6:31177674	5′ Flanking, exon 1	C>T	0.6761	0.7065	0.31310	0.9706	0.3779	0.5895
*PSORS1C3/rs3871246*	6:31177654	5′ Flanking, exon 1	A>G	0.3592	0.3370	0.41034	0.3418	0.3410	0.9675
*PSORS1C3/rs11967629*	6:31177636	Splice region variant	G>A	0.3099	0.2500	0.21685	0.8061	0.0777	0.7716
*PSORS1C3/+280*	6:31177647	Intron	G>A	0.1620	0.0652		0.2655	0.8941	0.3101
*CARD14/rs11653893*	17:80205031	Intron	A>G	0.5000	0.5000	0.35863	0.2051	0.3372	0.8987
*CARD14/rs189286068*	17:80205039	Synonymous	C>T	0.0282	0.0326	0.00279	0.9706	0.9742	0.9459
*CARD14/c.3150 p.T812A/S*	17:80205070	Missense	A>G/T	0.0211	0.0213		0.9927	0.9972	0.9892
*CARD14/rs11652075*	17:80205094	Missense	C>T	0.5845	0.5000	0.35304	0.5550	0.3372	0.5725
*CARD14/rs61757652*	17:80205117	Synonymous	C>T	0.1197	0.1739	0.10004	1.3132	0.3608	0.2066
*CARD14/rs1486223942*	17:80205169	synonymous	C>T	0.0141	0.0000	0.00001	0.9928	N/A	0.9957
*CARD14/c.3285+54*	17:80205259	Intron	C>G	0.0211	0.0000		0.9836	N/A	0.9902
*CARD14/rs376428578*	17:80205308	Intron	C>A	0.1901	0.3696	0.00006	0.1299	0.0004	0.0007
*TLR4/rs2149356*	9:117711921	Intron	T>G	0.7210	0.6850	0.47883	0,9928	0,5624	0.6785
*TLR4/c.331-428*	9:117711961	Intron	T>G	0.1360	0.1520		0.4219	0.4767	0.2029
*TLR4/c.331-200*	9:117712189	Intron	G>A	0.0143	0.0109		0.9927	0.9972	0.9901
*TLR4/c.331-102*	9:117712277	Intron	T>A	0.0357	0.0109		0.9531	0.9972	0.9599
*TLR4/c.331-1*	9:117712388	Intron	G>C	0.0143	0.0109		0.9927	0.9972	0.9901
*TLR4/rs770576183*	9:117712401	Missense	G>C	0.0210	0.0000	0.00001	0.0000	N/A	0.0000
*TLR4/rs1018673641*	9:117712426	Missense	A>T	0.0790	0.0330		0.7753	0.9742	0.7873
*TLR4/c.371 p.L101 L*	9:117712429	Synonymous	C>T	0.1571	0.0978		0,07831	0,2105	0,01651
*TLR4/c.376 p.S102S*	9:117712434	Synonymous	C>T	0.1214	0.0217		0.5124	0.9887	0.6333

Position refers to the GRCh38.p10 assembly; MAF: Minor allele frequency; HWE: Hardy-Weinberg equilibrium was checked by Chi-squared test; N/A: Not available; 1000G MAF: MAF data from 1000 genomes project.

**Table 2-:** Comparison of genotype frequencies of TLR4, CARD14 and PSORS1C3 variants between psoriatic patients and controls.

SNP	Gene	Test model	Controls (n=46)	Patients (n=71)	OR	95% CI	p-Value
rs887464	*PSORS1C3*	GG	20 (43.48%)	32 (45.07%)			
		GA	24 (52.17%)	21 (29.58%)	0.5469	0.2434 - 1.2286	0.065^(2)^
		AA	2 (4.35%)	18 (25.35%)	5.625	1.1772 - 26.8779	0.001^(1)^
rs3868542	*PSORS1C3*	AA	18 (39.13%)	30 (42.25%)			
		AG	22 (47.83%)	28 (39.44%)	0.7636	0.3403 - 1.7136	0.44^(2)^
		GG	6 (13.04%)	13 (18.31%)	1.3	0.4199 - 4.0250	0.673^(1)^
rs11507945	*PSORS1C3*	CC	23 (50%)	33 (46.48%)			
		CT	23 (50%)	30 (42.25%)	0.9091	0.4249 - 1.9451	0.772^(2)^
		TT	0 (0%)	8 (11.27%)	11.9254	0.6558 - 216.8568	0.001^(1)^
rs3871247	*PSORS1C3*	CC	18 (39.13%)	29 (40.85%)			
		CT	25 (54.35 %)	29 (40.85%)	0.72	0.3251 - 1.5944	0.292^(2)^
		TT	3 (6.52%)	13 (18.3%)	2.6897	0.6724 - 10.7591	0.673^(1)^
rs369029873	*PSORS1C3*	GG	27 (58.7%)	47 (66.2%)			
		GA	19 (41.3%)	24 (33.8%)	0.7256	0.3374 - 1.5605	0.381^(2)^
rs3130506	*PSORS1C3*	CC	2 (4.35%)	7 (9.86%)			
		CT	23 (50%)	32 (45.07%)	0.3975	0.0756 - 2.0913	0.151^(2)^
		TT	21 (45.65%)	32 (45.07%)	0.4354	0.0824 - 2.3015	0.157^(2)^
rs3871246	*PSORS1C3*	AA	18 (39.13%)	32 (45.07%)			
		AG	25 (54.35%)	27 (38.03%)	0.6075	0.2748 - 1.3431	0.131^(2)^
		GG	3 (6.52%)	12 (16.9%)	2.25	0.5600 - 9.0400	0.163^(1)^
rs11967629	*PSORS1C3*	GG	23 (50%)	35 (49.3%)			
		GA	23 (50%)	28 (39.44%)	0.8	0.3733 - 1.7145	0.467^(2)^
		AA	0 (0%)	8 (11.27%)	11.2535	0.6195 - 204.4117	0.002^(1)^
+280	*PSORS1C3*	AA	40 (86.96%)	48 (67.61%)			
		AG	6 (13.04%)	23 (32.39%)	3.1944	1.1850 - 8.6112	0.002^(2)^
rs11653893	*CARD14*	AA	14 (30.43%)	14 (19.72%)			
		AG	18 (39.13%)	43 (60.56%)	2.3889	0.9494 - 6.0112	0.023^(2)^
		GG	14 (30.43%)	14 (19.72%)	1	0.3508 - 2.8510	1^(1)^
rs189286068	*CARD14*	CC	43 (93.48%)	67 (93.37%)			
		CT	3 (6.52%)	4 (6.63%)	0.8557	0.1825 - 4.0124	1^(1)^
c.3150 p.T812A/S	*CARD14*	AA	45 (97.83%)	68(95.77%)			
		AG	1 (2.17%)	2 (2.82%)	1.3235	0.1165 - 15.0318	0.683^(1)^
		AT	0	1 (1.41%)	0.6618	0.0403 - 10.8535	0.497^(1)^
rs11652075	*CARD14*	CC	14 (30.43%)	17 (23.94%)			
		CT	18 (39.13%)	40 (56.33%)	1.8301	0.7441 - 4.5008	0.124^(2)^
		TT	14 (30.43%)	14 (19.72%)	0.8235	0.2957 - 2.2936	0.694^(1)^
rs61757652	*CARD14*	CC	30 (65.22%)	54 (76.06%)			
		CT	16 (34.78%)	17 (23.94%)	0.5903	0.2611 - 1.3344	0.121^(1)^
rs1486223942	*CARD14*	CC	46 (100%)	69 (97.18%)			
		CT	0	2 (2.82%)	3.3453	0.1570 - 71.2792	0.246^(1)^
c.3285+54	*CARD14*	CC	46 (100%)	68 (95.77%)			
		CG	0	3 (4.23%)	4.7518	0.2398 - 94.1681	0.121^(1)^
rs376428578	*CARD14*	CC	12 (26.09%)	44 (61.97%)			
		CA	34 (73.91%)	27 (38.03%)	0.2166	0.0960 - 0.4888	<0.001^(2)^
rs2149356	*TLR4*	TT	3 (6.52%)	6 (8.45%)			
		TG	23 (50%)	30 (42.25%)	0.6522	0.1472 - 2.8897	0.781^(2)^
		GG	20 (43.48%)	35 (49.3%)	0.875	0.1970 - 3.8858	1^(2)^
c.331-428	*TLR4*	TT	32 (69.57%)	51 (71.83%)			
		TG	14 (30.43%)	20 (28.17%)	0.8964	0.3973 - 2.0221	0.876^(1)^
c.331-200	*TLR4*	GG	45 (97.83%)	68 (95.77%)			
		GA	1 (2.17%)	3 (4.23%)	1.9853	0.2002 - 19.6899	0.683^(1)^
c.331-102	*TLR4*	TT	45 (97.83%)	66 (92.96%)			
		TA	1 (2.17%)	5 (7.04%)	3.4091	0.3853 - 30.1654	0.088^(1)^
c.331-1	*TLR4*	GG	45 (97.83%)	68 (95.77%)			
		GC	1 (2.17%)	3 (4.23%)	1.9853	0.2002 - 19.6899	0.683^(1)^
rs770576183	*TLR4*	GG	46 (100%)	69 (97.18%)			
		GC	0 (0%)	1 (1.41%)	2.0072	0.0800 - 50.3437	0.495^(1)^
		GA	0 (0%)	1(1.41%)	2.0072	0.0800 - 50.3437	0.495^(1)^
rs1018673641	*TLR4*	AA	44 (95.65%)	60 (84.5%)			
		AT	2 (4.35%)	11 (15.5%)	4.0333	0.8509 - 19.1187	0.008^(1)^
c.371 p.L101 L	*TLR4*	CC	27 (58.7%)	41 (57.75%)			
		CT	19 (41.3%)	30 (42.25%)	1.0398	0.4899 - 2.2067	1^(2)^
c.376 p.S102S	*TLR4*	CC	20 (43.48%)	40 (56.34%)			
		CT	26 (56.52%)	31 (43.66%)	0.5962	0.2821 - 1.2598	0.089^(2)^

*P*-values were calculated by either Fisher’s exact test ^(1)^ or Chi-squared test ^(2)^; p < 0.05 (in bold) indicates statistical significance from healthy donors; OR: Odds ratio; 95% CI: 95% confidence interval of odds ratio.

**Figure 2-:**
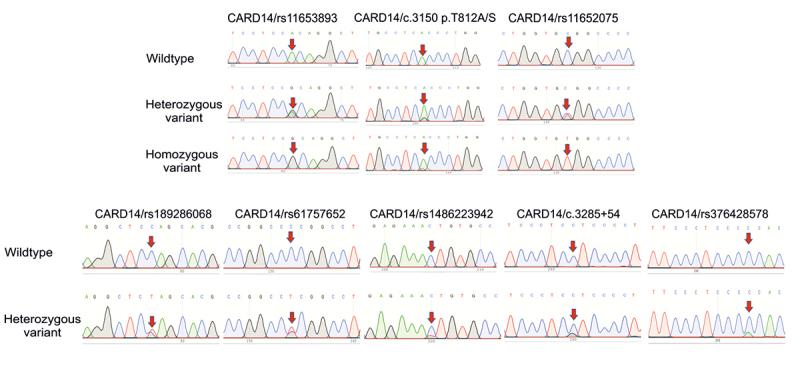
Polymorphisms of CARD14 gene in psoriatic patients and controls. Partial sequence chromatograms of *CARD14* gene from wildtype (1^st^ panels) and variant (2^nd^ and 3^rd^ panels) genotypes of the rs11653893 A>G, p.T812A/S (c.3150 A>G/T), rs11652075 C>T, rs189286068 C>T, rs61757652 C>T, rs1486223942 C>T, c.3285+54 C>G and rs376428578 C>A polymorphisms are shown. Arrows indicate the location of the base changes.

Next, sequencing of *CARD14* gene determined 8 nucleotide changes, including a SNP rs11653893 A>G in intron 20; 02 SNPs c.3285+54 C>G and rs376428578 C>A in intron 21; 02 non-synonymous SNPs (nsSNPs) p.T812A/S (c.3150 A>G/T) and rs11652075 C>T; and 3 synonymous SNPs rs189286068 C>T, rs61757652 C>T and rs1486223942 C>T in exon 21 (Tables 1, 2 and Figure 2). The genotype distribution of the 8 SNPs, except for the intronic SNP rs376428578 was in accordance with HWE (p > 0.05) (Table 1). The MAF for the SNP rs61757652 was slightly lower, whereas the MAF for the 2 SNPs rs1486223942 and c.3285+54 was higher in psoriatic patients than the control group. Among these 2 SNPs, the CT genotype of the snSNP rs11652075 was slightly higher in cases than controls with the carrier frequencies of 56.33% and 39.13%, respectively (Table 2). In addition, the AG genotype of the intronic SNP rs11653893 had a higher prevalence in cases, however it was not a risk factor for psoriasis as predicted by using the SD-Score or MaxEntScan predictor program.

**Figure 3-:**
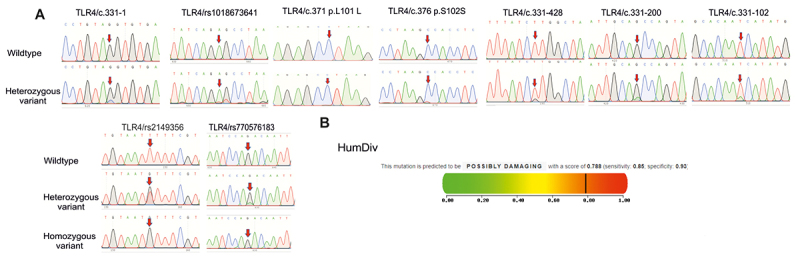
Polymorphisms of TLR4 gene in psoriatic patients and controls. **A**: Partial sequence chromatograms of *TLR4* gene from wildtype (1^st^ panels) and variant (2^nd^ and 3^rd^ panels) genotypes of the rs2149356 T>G, c.331-428 T>G, c.331-200 G>A, c.331-102 T>A, c.331-1 G>C, rs770576183 G>C/A, rs1018673641 A>T, p.L101L (c.371 C>T) and p.S102S (c.376 C>T) polymorphisms are shown. Arrows indicate the location of the base changes. **B**: Functional prediction of the SNP rs1018673641 by the Polyphen-2 program.

Finally, sequencing of *TLR4* gene identified 9 nucleotide changes, including 5 SNPs rs2149356 T>G, c.331-428 T>G, c.331-200 G>A, c.331-102 T>A and c.331-1 G>C in intron 3 and 4 exonic SNPs rs770576183 G>C/A, rs1018673641 A>T, p.L101L (c.371 C>T) and p.S102S (c.376 C>T) in exon 4 (Tables 1, 2 and Figure 3A). Among the 9 SNPs found in *TLR4* gene, the 2 SNPs rs770576183 G>C/A and rs1018673641 A>T were nsSNPs and the 2 remaining exonic SNPs were silent. The genotype distribution of the 9 SNPs in *TLR4* gene was in agreement with the HWE (p > 0.05). Importantly, we noted that the MAF of the SNPs c.331-102 T>A, rs1018673641 A>T, p.L101L and p.S102S was significantly higher in the patient group compared to control group and the difference in the MAFs for the 5 remaining SNPs between the two groups was not observed (Table 1). 

For determination of susceptibility to psoriasis by evaluating the deleterious effect of the nsSNPs in *TLR4* gene, the results indicated that among the 2 nsSNPs, only the rs1018673641 was predicted to be probably damaging by Polyphen-2 with score of 0.788 (score range: 0-1; sensitivity: 0.85; specificity: 0.93) (Figure 3B). Accordingly, the rs1018673641 might be one of the most deleterious nsSNPs in *TLR4* gene. Moreover, AT genotype of the rs1018673641 showed higher frequency in cases (15.5%) compared to healthy individuals (4.35%; p = 0.008, Table 2), while the distribution of the rs770576183 genotype frequencies was similar in the two groups.

### Haplotype and linkage disequilibrium analysis of *PSORS1C3*, *CARD14* and *TLR4* variants

**Figure 4-:**
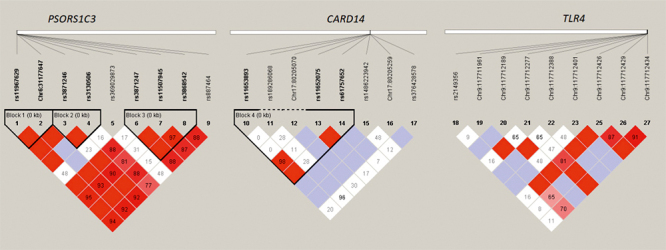
Linkage disequilibrium (LD) analysis of TLR4, CARD14 and PSORS1C3 genes in psoriatic patients and controls. Linkage disequilibrium analysis shows the genotyped variants in *PSORS1C3, CARD14* and *TLR4* genes. D’ value was shown in the LD block.

Last but not least, we tested the association of statistically inferred haplotypes with the risk of psoriasis. As shown in Figure 4, the SNPs in *PSORS1C3* gene formed three haplotype blocks and contributed to eighteen haplotypes in our study (Figure 4 and Table 3). Block 1 was found to include the two SNPs rs11967629 and +280 G>A; block 2 consisted of rs3130506 and rs3871246; and block 3 comprised rs3868542, rs11507945 and rs3871247. The SNPs in *CARD14* gene formed haplotype block 4, which consists of rs11653893, rs11652075 and rs61757652. 

**Table 3 -:** Comparison of haplotype frequencies of TLR4, CARD14 and PSORS1C3 variants between psoriatic patients and controls.

SNPs in haplotype block	Haplotype	Controls (n=46)	Patients (n=71)	p-Value
PSORS1C3/rs11967629 and +280 G>A				4-df, **P<0.001**
	G-G	23 (50%)	35 (49.3%)	
	GA-G	17 (36.96%)	7 (9.86%)	0.002
	A-G	0 (0%)	6 (8.45%)	0.007
	A-AG	0 (0%)	2 (2.82%)	0.243
	GA-GA	6 (13.04%)	21 (29.6%)	0.029
PSORS1C3/rs3130506 and rs3871246				5-df, P=0.057
	C-A	2 (4.35%)	7 (9.86%)	
	CT-A	11 (23.91%)	18 (25.35%)	0.229
	CT-AG	13 (28.26%)	14 (19.72%)	0.07
	T-AG	12 (26.09%)	12 (16.9%)	0.063
	T-A	5 (10.87%)	7 (9.86%)	0.296
	T-G	3 (6.52%)	13 (18.3%)	1
PSORS1C3/rs3868542, rs11507945 and rs3871247				6-df, **P=0.007**
	A-C-C	18 (39.13%)	28 (39.44%)	
	AG-C-CT	3 (6.52%)	5 (7.04%)	1
	G-C-T	2 (4.35%)	1 (1.41%)	0.361
	G-CT-T	2 (4.35%)	4 (5.63%)	0.739
	G-T-T	0 (0%)	8 (11.27%)	0.002
	AG-CT-CT	21 (45.65%)	24 (33.8%)	0.425
	A-CT-CT	0 (0%)	1 (1.41%)	1
CARD14/rs11653893/rs11652075/rs61757652				8-df, **P<0.001**
	A-C-C	8 (17.4%)	7 (9.86%)	
	A-C-CT	6 (13.04%)	7 (9.86%)	0.774
	AG-C-C	0 (0%)	1 (1.41%)	0.393
	G-CT-C	0 (0%)	1 (1.41%)	0.393
	AG-T-C	0 (0%)	1 (1.41%)	0.393
	AG-C-CT	0 (0%)	2 (2.82%)	0.07
	AG-CT-C	8 (17.4%)	31 (43.67)	0.002
	AG-CT-CT	10 (21.74%)	8 (11.27%)	0.792
	G-T-C	14 (30.43%)	13 (18.3%)	1
TLR4/rs1018673641-c.371-c.376				5-df, **P<0.001**
	A-C-C	18 (39.13%)	39 (54.93%)	
	A-T-C	2 (4.35%)	1 (1.41%)	0.163
	A-T-T	15 (32.6%)	18 (25.35%)	0.069
	A-C-T	9 (19.56%)	2 (2.82%)	<0.001
	T-C-T	1 (2.18%)	1 (1.41%)	0.572
	T-T-T	1 (2.18%)	10 (14.08%)	0.047

*P*-values were calculated by Chi-squared test; p < 0.05 (in bold) indicates statistical significance from healthy donors.

There were five haplotypes within block 1. Using the common haplotype G-G as a reference, two haplotypes (A-G and GA-GA) were associated with increased risks of psoriasis (8.45% and 29.6% for patients vs. 0% and 13.04% for controls, *p*=0.007 and *p*=0.029, respectively), whereas another haplotype (GA-G) was significantly less frequent in cases compared with controls (36.96% vs. 9.86%, p=0.002). A total of six haplotypes were inferred within block 2, in which two haplotypes (CT-AG and T-AG) were associated with decreased risks of psoriasis (19.72% and 16.9% for patients vs. 28.26% and 26.09% for controls, *p*=0.07 and *p*=0.063, respectively). Block 3 had seven haplotypes and the wild-type haplotype A-C-C was used as a reference. The haplotype frequency of G-T-T in block 3 was 11.27% in cases, while it was completely absent in controls (*p*=0.002). In block 4, there were nine haplotypes. Using the wild-type haplotype A-C-C as a reference, one haplotype (AG-CT-C) was significantly associated with psoriasis susceptibility (43.67% vs. 17.4%, *p*=0.002) (Table 3). 

In addition, we also found significant differences between cases and controls for two haplotypes (A-C-T and T-T-T) of rs1018673641-c.371-c.376 in *TLR4* gene. The frequency of the T-T-T haplotype was significantly elevated (14.08% vs. 2.18%, *p*=0.047), whereas the A-C-T haplotype was significantly less frequent in cases compared with controls (19.56% vs. 2.82%, p<0.001) (Table 3).

Moreover, the linkage disequilibrium (LD) analysis showed a tight linkage between almost all the SNPs detected in *PSORS1C3* gene (except for the rs369029873 and rs887464 variants). Of the 8 variants in the *CARD14* gene, the three SNPs rs11653893, rs11652075 and rs61757652 were tightly linked. However, no haplotype block was found in the *TLR4* gene (Figure 4), probably because of the small sample size. 

### Association between inflammatory cytokines and genotypes in psoriatic patients

**Figure 5-:**
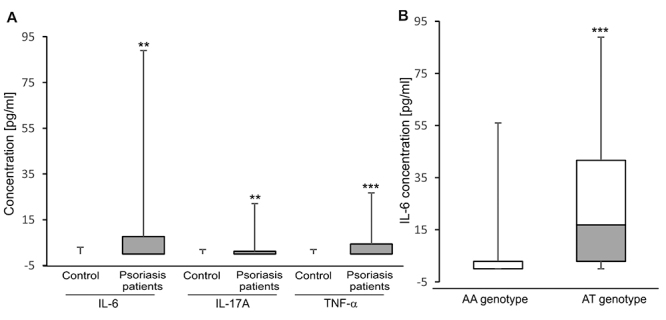
Expression of inflammatory cytokines in psoriatic patients. A: Arithmetic means ± SEM (*n* = 46-71) of IL-6, IL-17A and TNF-α concentrations are attained from sera of healthy donors and psoriatic patients. ** (p<0.01) and *** (p<0.001) indicate significant differences from healthy donors (Mann-Whitney U test). The box plots denote the median, IQR and minimum and maximum values. B: Arithmetic means ± SEM (*n* = 11-60) of IL-6 concentration is attained from sera of psoriatic patients carrying the AA or AT genotype of SNP rs1018673641 in *TLR4* gene. *** (p<0.001) indicates significant difference from the AA genotype (Mann-Whitney U test). The box plots denote the median, IQR and minimum and maximum values.

*TLR4* and *CARD14* are known as inducers of inflammatory reaction (Scudiero et al., 2011; Takeshita et al., 2017; Tang et al., 2021), thus unusual genotypes in these genes may be related to the release of cytokine production in psoriatic patients. Similar to a recent study (Christophers et al., 2019), we observed that levels of IL-6, IL-17A and TNF-α in psoriatic patients were found higher than control individuals (Figure 5A). Furthermore, the increased level of these cytokines was seen in severe psoriasis (Christophers *et al.,* 2019). As expected, level of IL-6 was significantly higher in patients carrying the AT genotype as compared with carriers of the AA genotype of SNP rs1018673641 in *TLR4* gene (Figure 5B). However, no significant difference between levels of IL17A and TNF-α and the SNPs in *PSORS1C3, CARD14* and *TLR4* genes was observed (data now shown). The evidence for the association pointed out that psoriatic patients carrying the rs1018673641 AT genotype in *TLR4* gene was sensitive to IL6-induced inflammatory response.

## Discussion

This study showed the increased risk of developing psoriasis in patients carrying SNPs in *PSORS1C3, CARD14* and *TLR4* genes (Holm et al., 2005; Smith et al., 2016; Danis et al., 2018). Unlike *PSORS1C3* gene, the functional study of *TLR4* and *CARD14* genes demonstrates that they play important roles in regulating pro-inflammatory gene expression through activation of intracellular signaling such as NF-κB or MAPK (Wang et al., 2018; Tang et al., 2021). Therefore, genetic variation of *TLR4* and *CARD14* genes might be the contributing risk factors for psoriasis by modulating the cellular physiological processes.

Little is known about the functional role of *PSORS1C3* gene in modulating inflammatory response, the association of *PSORS1C3* polymorphisms with psoriasis in various population has been well documented (Holm et al., 2005; Chang et al., 2006). Similar to the Chinese population (Chang *et al.,* 2006), we observed that frequencies of the 2 SNPs -26 C>T (rs3871247) and +246 A>G (rs3871246) in the *PSORS1C3* gene were comparable in the two groups, whereas they are previously reported to be susceptibility SNPs for psoriasis in Swedish population (Holm *et al.,* 2005). In addition, similar to a recent study by Holm *et al.* (2005), the presence of the SNP rs3868542 in *PSORS1C3* gene was unaffected patients with psoriasis. Moreover, the present study demonstrated for the first time that, of the 4 remaining genotyped SNPs in the 5’ flanking region, all the rs887464 AA, rs11507945 TT, rs11967629 AA and +280 GA genotypes were prominently associated with psoriasis. The evidences noted that disease susceptibility SNPs in the *PSORS1C3* gene were different from one population to another.

The regulatory role of inflammatory skin disorders is reported mediated through CARD14 signaling. Recently, the SNP rs11653893 in the *CARD14* is detected in patients with pityriasis rubra pilaris (Gal et al., 2019), a rare form of psoriatic skin disease. Similarly, we observed that carriers of the AG genotype were detected at a higher frequency compared to patients carrying the AA or GG genotype in SNP rs11653893. A recent report by Tsoi et al. (2012) reveals that the SNP rs11652075 in *CARD14* gene is sensitive with psoriasis in Caucasian population, while we observed an increased frequency of the rs11652075 CT genotype in patient group, but not reaching to statistical significance (*p*=0.124).

Similar to *CARD14* gene, the role of *TLR4* gene is related to the development of a number of inflammatory diseases, such as psoriasis (Smith et al., 2016). Among the 9 SNPs in *TLR4* gene observed, the rs1018673641 was found to be most likely to exert deleterious effect. Role of TLR4 is especially important in mediating chronic inflammatory condition (Takeshita et al., 2017). Expression of TLR4 is enhanced in the epidermis of psoriatic skin (Shao et al., 2019), leading to infiltration of Th17 cells and their activation (Tang et al., 2021). The release of cytokines, such as IL-1β (Shepherd et al., 2004) and TNF-α/IL-23/IL-17 (Cai et al., 2019) by TLR4-mediated inflammatory immune cells is related to the severity of psoriasis (Ren et al., 2020). Besides, several SNPs in *TLR4* gene have been reported associated with psoriasis susceptibility (Smith *et al.,* 2016), we additionally indicated that the AT genotype of the SNP rs1018673641 in *TLR4* gene could be the high-risk genotype for psoriasis.

Interestingly, we demonstrated that all the three haplotypes (A-G, GA-GA and G-T-T) in *PSORS1C3* gene, the AG-CT-C haplotype in *CARD14* gene and the T-T-T haplotype in *TLR4* gene were detected at higher frequencies in patients with psoriasis compared to controls (Table 3). Unlike *PSORS1C3* gene, reports on the effects of *CARD14* and *TLR4* haplotypes in psoriasis susceptibility are limited (Sugiura et al., 2014; Traks et al., 2015). The haplotypes found in *PSORS1C3, CARD14* and *TLR4* genes were reported for the first time in this finding. 

Finally, we observed enhanced expression of inflammatory cytokines IL-6, IL-17A and TNF-α in sera of psoriatic patients. Similarly, the increased level of these cytokines is seen in severe psoriasis (Christophers et al., 2019). More importantly, level of IL-6 was found higher in carriers of the AT genotype as compared to patients carrying the AA genotype of SNP rs1018673641 in *TLR4* gene. As TLR4 is known to play a crucial role in the regulation of immune response, an investigation in a mouse model showed that serum cytokine productions secreted from immune cells are defected in *TLR4*-deficient mice (Hollingsworth et al., 2006). A recent study indicates that psoriasis-like inflammation damages the renal function via the TLR4-mediated IL-6 production in mice (Ren et al., 2020). In this work, we predict that IL-6-mediated inflammation in psoriasis could be involved in variant *TLR4* genotypes. 

There are several potential limitations in the current study. The sample size was not sufficient for statistical measurements to verify significant relationship between the SNPs in *PSORS1C3, CARD14* and *TLR4* genes and psoriasis susceptibility in the Vietnamese population. Besides, further functional research is necessary for investigating the regulatory effects of the SNP rs1018673641 in *TLR4* gene on inflammatory response in psoriasis. 

In conclusion, the deleterious effect of the SNP rs1018673641 in *TLR4* gene could partially contribute to chronic inflammation in psoriasis and be a good candidate for further study on its role in regulating functional activation of immune cells in psoriatic patients. 
